# Mesoscopic Inhomogeneities in Ethanol–Water Mixtures: Are They Nanobubbles, Impurity Aggregates, or Nanoscale Gas–Water Composite Structures?

**DOI:** 10.1021/acs.langmuir.5c05369

**Published:** 2026-02-10

**Authors:** Chien-Chun Chen, Wei-Hao Hsu, Chun-Jen Chen, Tzu-Chieh Yen, Ching-Hsiu Chen, C. K. Chan, Che-Ming Jack Hu, Ing-Shouh Hwang

**Affiliations:** † 71551 Institute of Physics, Academia Sinica, Nankang, Taipei 115, Taiwan; ‡ Institute of Biomedical Sciences, Academia Sinica, Taipei 115, Taiwan

## Abstract

The study of bulk nanobubbles is a rapidly expanding field, and mixing water with alcohol has been proposed as a simple method for generating such structures. However, previous light-scattering investigations of alcohol–water mixtures have produced inconsistent and often contradictory results. In this work, we employed static light scattering (SLS), dynamic light scattering (DLS), nanoparticle tracking analysis (NTA), and transmission electron microscopy (TEM) to examine ethanol–water mixtures prepared from two distinct ethanol sources. Our findings indicate that the strongly light-scattering objects observed in these mixtures originate from impurities in the ethanol. These impurities could not be removed by degassing alone but were effectively eliminated through ethanol purification methods such as distillation. NTA measurements revealed high concentrations of dim, colloidal-like particles in mixtures prepared with high-purity ethanol. These particles could be removed through vacuum degassing, suggesting that their presence is linked to gas supersaturation. Based on our observations, we propose that the impurities likely originate from additive molecules leached from plastic containers used to store ethanol. Upon mixing with water, these molecules may aggregate into nanoparticles, contributing to the strong scattering signals. In contrast, the dim colloidal-like particles appear to be gas-containing nano-objects, most likely mesoscopic clathrate hydrate structures, as confirmed by TEM imaging. Our results provide a coherent explanation that reconciles the seemingly contradictory findings in the literature.

## Graphical abstract

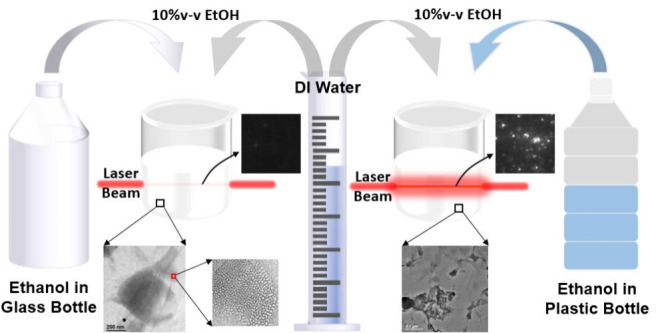

## Introduction

In recent years, research on bulk nanobubbles—gas-filled structures of mesoscopic scale (typically tens to hundreds of nanometers) suspended in aqueous solutions—has grown rapidly. Despite numerous demonstrated technical applications and the emergence of commercial products claiming to utilize them, bulk nanobubbles remain a profound scientific mystery.^1–4^ A central unresolved question is their extraordinary stability, with reported lifetimes ranging from hours to even month.^2–4^ According to the Young–Laplace equation (Δ*P* = 2γ/*r*), the internal pressure of a gas bubble increases inversely with its radius. At nanometer-scale dimensions, this pressure should, in principle, be so high that the bubble cannot remain stable for more than 100 ms. To date, no theoretical framework has satisfactorily explained this long-term stability,^4^ casting doubt on whether bulk nanobubbles genuinely exist in the solutions where they are claimed to be present. Among the various methods proposed for generating bulk nanobubbles, one of the simplest involves mixing water with monohydric alcohols such as methanol or ethanol.^5–8^ Light scattering techniques—including static light scattering (SLS), dynamic light scattering (DLS), and nanoparticle tracking analysis (NTA)—have revealed colloidal-sized structures on the order of ∼ 100 nm in water–alcohol mixtures at low alcohol concentrations.^5–13^ Given that water and alcohol are fully miscible across all proportions, the presence of mesoscopic structures significantly larger than the molecular dimensions of either component is unexpected and intriguing. Some researchers interpret these colloidal-sized entities as bulk nanobubbles, citing their sensitivity to dissolved gas concentrations as supporting evidence.^5–8^ However, other experimental findings challenge this interpretation, suggesting that the observed structures may not be gas bubbles at all.^10–13^

Sedlák et al. were the first to report the presence of mesoscopic structures in mixtures of water with small water-miscible molecules—such as alcohols and urea—using DLS.^9^ They attributed these structures to the association of small molecules with water, forming supramolecular structures or complexes. In 2007, Jin et al. investigated aqueous solutions containing small organic solvents, including ethanol, and identified a slow relaxation mode corresponding to light-scattering entities approximately 100 nm in size.^5^ These nano-objects were removed through repeated filtration but reappeared upon the injection of filtered air. Based on this behavior, Jin et al. proposed that the structures were bulk nanobubbles stabilized by organic molecules adsorbed at the gas–water interface. Contrastingly, Häbich et al. argued that the observed light-scattering entities were not nanobubbles but rather water-insoluble impurities.^10^ They reported strong scattering signals in aqueous solutions of ethanol without any treatment as well as in aqueous solutions of gassed or degassed ethanol, while no such signals were detected in solutions prepared from distilled ethanol. Qiu et al., however, supported the nanobubble hypothesis. Using nanoparticle tracking analysis (NTA), they found that the concentration of mesoscale objects was reduced 5-fold when degassed water and degassed ethanol were mixed, suggesting the presence of bulk nanobubbles.^6^ Alheshibri and Craig employed resonant mass measurement to determine a density of approximately 0.91 g/cm^3^ for the mesoscopic structures in ethanol–water (EW) mixtures.^13^ They also demonstrated that these structures were incompressible under pressures ranging from 1 to 5 atm—findings inconsistent with the behavior expected of bulk nanobubbles. Furthermore, they observed a significant decrease in the concentration of mesoscopic structures when degassed ethanol and degassed water were mixed, concluding that the nanoparticles likely result from the accumulation of contaminants at the interface of dissolving bubbles.^13^ Rak et al. similarly attributed the light-scattering objects in EW mixtures to nanoscale segregation of hydrophobic contaminants in ethanol, because no such objects were present when ethanol was purified prior to mixing with water.^12^ In contrast, Jadhav and Barigou applied a range of physical and chemical analytical techniques to demonstrate that the nano-objects formed via various methods—including water–ethanol mixing—were gas-filled domains, not solid or liquid contaminants.^7^ They concluded that bulk nanobubbles do exist and exhibit stability. To date, the debate over whether the mesoscopic structures observed in ethanol–water mixtures are genuine nanobubbles or merely impurities remains unresolved.^8,14,15^

To address the conflicting findings reported in the literature, we employed static and dynamic light scattering, along with nanoparticle tracking analysis (NTA), to re-examine ethanol–water (EW) mixtures containing 10% and 20% ethanol by volume. Two different ethanol sources—Sigma-Aldrich and Baker Analyzed—were used, yielding markedly different results. Strongly scattering objects were consistently detected in EW mixtures prepared with ethanol from Sigma-Aldrich, and these could not be effectively removed by degassing. In contrast, no such objects were detected when Baker Analyzed ethanol was used, or when Sigma-Aldrich ethanol was distilled prior to mixing with water. Using NTA, we identified a high concentration of dim nanoparticles in mixtures prepared with Baker Analyzed ethanol. These particles were eliminated either by vacuum degassing or by predegassing both water and ethanol before mixing. We employed transmission electron microscopy (TEM) on EW mixtures encapsulated within graphene liquid cells (GLCs),^16^ which allow direct visualization of liquid-phase structures with subnanometer to atomic resolution—far surpassing the capabilities of optical techniques. TEM analysis confirmed that EW mixtures prepared with ethanol from Sigma-Aldrich contained impurities, which were seldom observed when the ethanol had been stored in a glass bottle for several weeks. These findings may help reconcile previously conflicting reports regarding the presence of nanobubbles in alcohol–water mixtures and suggest a new direction for investigating the physicochemical nature of such systems.

## Experimental Section

### Materials and Sample Preparation

EW mixtures were prepared by mixing ethanol with pure water at volume ratios of 1:9 and 1:4 to yield 10% and 20% EW mixtures, respectively. Water and ethanol were carefully measured and separately transferred into either a glass beaker or a polypropylene (PP) centrifuge tube. The mixture was gently shaken for 30–40 s and subsequently left to stand for at least 5 min before further experimental procedures. For SLS and DLS measurements, deionized water with a resistivity of 18.2 MΩ·cm, produced using a Milli-Q system (Millipore Corp.), was used. For NTA, commercially available sterilized distilled water (NANG KUANG PHARMACEUTICAL CO. Ltd.) served as the water source. In most experiments, ethanol from BAKER ANALYZED (A.C.S. Reagent, CAS NO: 64–17–5, 4L, container: glass, purity >99.9%) and Sigma-Aldrich (puriss. p.a., absolute, 2.5L, container: high-density polyethylene (HDPE), purity >99.8%) was used without further purification. In select experiments, Sigma-Aldrich ethanol was distilled once prior to mixing with water. We employed a standard distillation procedure commonly used in chemistry laboratories. Sigma-Aldrich ethanol was transferred into a distillation flask and heated using a hot plate. As the temperature increased, ethanol vapor rose and passed through the condenser, where it was cooled by circulating water. The condensed ethanol was then collected in a clean glass beaker. Degassed water, ethanol, and EW mixtures were prepared by placing the respective liquids in a desiccator, evacuating to approximately 0.1 atm using an oil-free vacuum pump (Rocker 410, Rocker), and storing them under vacuum for more than 8 h. The desiccator was opened immediately before use to minimize reabsorption of dissolved gases. Unless otherwise specified, all experiments were conducted at room temperature (22–25 °C).

### Laser Light Scattering

SLS and DLS measurements were performed using Brookhaven Instruments BI-200SM goniometer system equipped with a mini-L30 laser (637 nm, 30 mW) and a BI-DS photomultiplier tube (PMT). Measurements were performed at a scattering angle of 90° to minimize reflections from the sample wall. During the experiments, scattering intensities were adjusted by tuning the incident laser power and scattering volume to ensure that the count rate was at least ten times higher than the PMT dark count. For comparison across different samples, scattering intensities were recorded under identical laser intensity and scattering volume conditions. SLS measurements were obtained as time-averaged count rates of scattered light from the DLS experiments, using the same scattering geometry and laser settings. These SLS intensities were then used to compare scattering behavior between samples. DLS detects fluctuations in the intensity of scattered light arising from the Brownian motion of particles, emulsions, bubbles, or molecules suspended in a liquid medium. Analysis of the time-dependent autocorrelation function of these fluctuations yields the translational diffusion coefficient, which is used to calculate the hydrodynamic radius via the Einstein–Stokes equation. It is important to note that DLS does not provide information on particle number density.

### NTA

All NTA measurements were conducted using a NanoSight NS500 instrument (Malvern, software version 3.1) at room temperature, equipped with a violet laser (70 mW, 405 nm). NTA tracks the Brownian motion of individual nanoparticles in suspension via laser light scattering microscopy. By applying the Einstein–Stokes equation to the observed particle trajectories and count rates, NTA yields both the hydrodynamic radius distribution and the number density of particles in the sample. In NTA measurements, image brightness is controlled by selecting the appropriate camera level. The camera level was increased until all particles in the sample were clearly visible, while ensuring that no more than 10% of the particles appeared saturated. Each camera level corresponds to a defined combination of gain and shutter time (Supporting Table 1 of the Supporting Information).

### TEM

The preparation protocols for graphene liquid cells (GLCs) have been detailed in previous studies.^17–19^ All GLCs were imaged using a field-emission TEM (JEM-2100F, JEOL) operated at an acceleration voltage of 100 kV. The background pressure was ∼ 5 × 10^–6^ Pa. Unless otherwise noted, bright-field TEM imaging was performed at underfocus to achieve high image contrast.

## Results and Discussion

### Laser Light Scattering of EW Mixtures

As shown in Figure 1, laser light scattering measurements showed very low scattering intensities (∼10K counts/s) for deionized (DI) water and pure ethanol, corresponding to the background detection limit of our system. In contrast, a markedly higher scattering intensity (∼125 K counts/s) was observed for a 20% EW mixture prepared using as-received ethanol from Sigma-Aldrich (denoted as 20% EW-Sigma). Notably, when the Sigma-Aldrich ethanol was distilled prior to mixing (20% EW-distilled Sigma), the scattering intensity decreased to near-background levels, consistent with the findings reported by Habich et al.^10^ Similarly, 20% EW mixtures prepared using as-received ethanol from Baker Analyzed (20% EW-Baker) also exhibited low scattering intensities, comparable to the background. Multiple independent measurements confirmed these results: only the 20% EW-Sigma sample consistently produced elevated scattering signals, while all other samples—including DI water, pure ethanol, 20% EW-distilled Sigma, and 20% EW-Baker—remained at background levels. These observations suggest the presence of strongly scattering objects exclusively in the 20% EW-Sigma mixture, likely due to a higher concentration of contaminants in the as-received Sigma-Aldrich ethanol compared to Baker Analyzed ethanol. Figure S1 presents the particle size distribution for the 20% EW-Sigma sample, indicating that the majority of detected particles fall within the 10–100 nm range.

**Figure 1:**
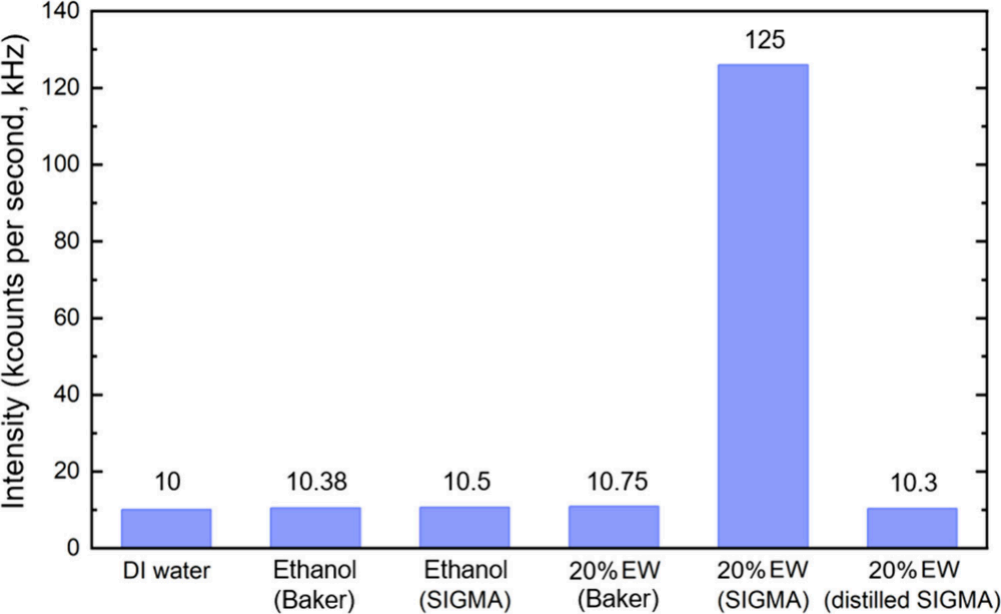
SLS measurements of deionized water, two commercially available ethanol sources, and 20% EW mixtures prepared using different protocols.

### NTA of EW Mixtures

Consistent with DLS measurements, NTA revealed a markedly higher concentration of strongly light-scattering particles in EW mixtures prepared using ethanol from Sigma-Aldrich, whereas only weakly scattering particles were detected in mixtures prepared with Baker Analyzed ethanol (Figure 2). A representative NTA image of 20% EW-Sigma acquired at camera level 12 is shown in Figure 2a, displaying a dense population (>5 × 10^9^ particles/mL) of bright particles. After degassing, the particle concentration in 20% EW-Sigma decreased by approximately 40%, yet a substantial number of bright particles remained visible (Figure 2b). In contrast, few particles were observed in 20% EW-Baker at camera level 12 (data not shown). However, when the camera level was increased to 15—corresponding to an image gain approximately 2.5 times greater than that at level 12—a significant concentration (∼2 × 10^9^ particles/mL) of dim particles became apparent (Figure 2c). For improved visualization, the brightness of Figure 2c is digitally enhanced by 40%. Upon degassing, most of these dim particles in 20% EW-Baker disappeared (Figure 2d), indicating that they likely represent gas-containing nano-objects.

**Figure 2:**
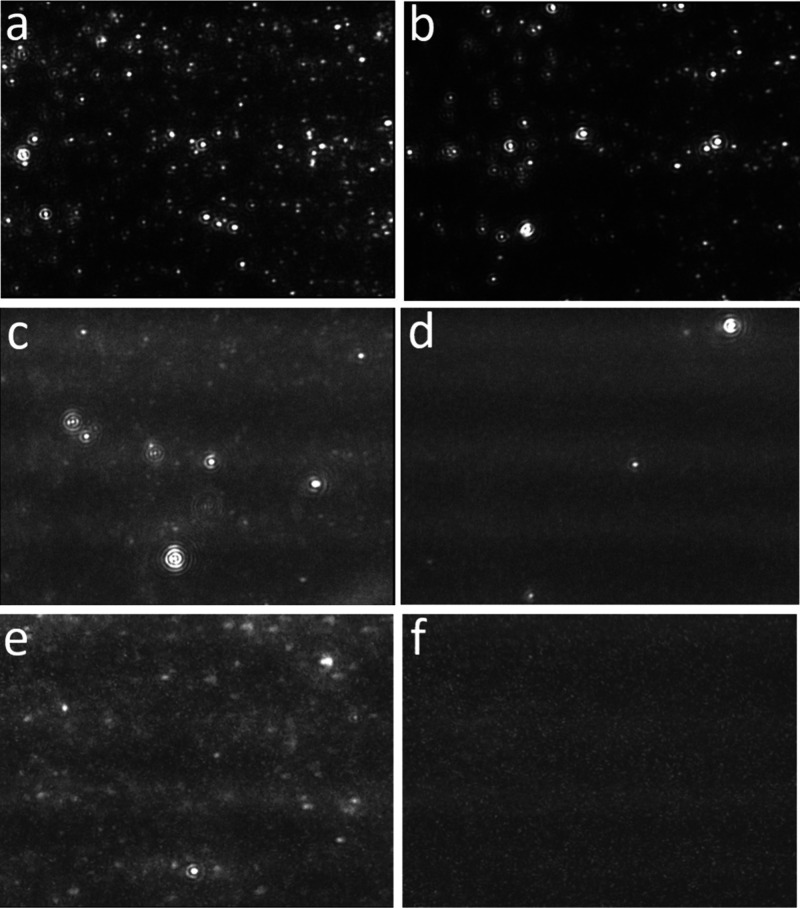
NTA images of EW mixtures. (a) 20% EW-Sigma; camera level of 12; detection threshold of 5. (b) 20% EW-Sigma after degassing; camera level of 12; detection threshold of 5. (c) 20% EW-Baker; camera level of 15; detection threshold of 4. The brightness is further enhanced by 40%. The particle concentration was ∼2 × 10^9^ particles/mL. (d) 20% EW-Baker after degassing; camera level of 15; detection threshold of 5. The particle concentration was 4 × 10^7^ particles/mL. The brightness is further enhanced by 40%. (e) 10% EW-Baker; camera level of 16; detection threshold of 5. The particle concentration was ∼1 × 10^9^ particles/mL. (f) 10% EW-Baker after degassing; camera level of 16; detection threshold of 5. The particle concentration was 3 × 10^7^ particles/mL.

In Figure 2a–c, the particle count per frame exceeded 200, which impeded reliable particle tracking and subsequent quantitative analysis. We thus reduced the ethanol concentration and prepared 10% EW-Sigma and 10% EW-Baker samples, each exhibiting particle concentrations of approximately 1 × 10^9^ particles/mL. At camera level 14 and 16, numerous bright particles were consistently observed in 10% EW-Sigma (Figure S2a,b), whereas 10% EW-Baker exhibited predominantly dim particles (Figure S2c,d). A representative NTA image of 10% EW-Baker acquired at camera level 14 is shown in Figure S2c, where only a few light-scattering particles are discernible. Increasing the camera level to 16 enhanced the visibility of dim particles in 10% EW-Baker; however, this also introduced visual artifacts such as noise and window stains (Figure 2e and Figure S2d). At camera level 16, Supporting Video 1 of the Supporting Information provides dynamic contrast between mobile dim particles undergoing Brownian motion and stationary stains, which are difficult to distinguish in static images. We conducted over 20 independent NTA measurements of 10% EW-Baker, consistently detecting a particle concentration of ∼ 1 × 10^9^ particles/mL with a size distribution ranging from 50 to 120 nm.^18^ Following vacuum degassing (10% EW-Baker-degassed), the dim particles nearly vanished (Figure 2f).^18^ These degassing experiments revealed that the concentration of strongly light-scattering particles in EW-Sigma (Figure 2b) decreased only modestly—by a few tens of percent—while the dim particles in EW-Baker were almost entirely eliminated (Figure 2d,f). Taken together, these observations suggest that the dim particles in EW-Baker are gas-containing nano-objects, whereas the strongly light-scattering particles in EW-Sigma may originate from mesoscopic contaminants or impurities.

According to Mie theory, the light scattering cross section depends on particle size and the refractive indices (RIs) of both the scattering particles and the surrounding medium, provided the wavelength and detection angle are specified. For particles of identical size, those exhibiting a greater RI contrast with the medium scatter light more intensely than those with a smaller RI difference. Our previous NTA study demonstrated that monodisperse polystyrene nanoparticles (RI ≈ 1.62) suspended in degassed 10% EW-Baker exhibit significantly stronger scattering than the dim particles of comparable size in the same medium.^18^ The study also indicates that the RI of the dim particles is approximately 1.27 at a laser wavelength of 405 nm, which is close to that of pure water (RI ≈ 1.34 at the same wavelength). Importantly, these dim colloidal-like particles cannot be gas bubbles. If they were, their scattering intensity would exceed that of polystyrene nanoparticles of the same size due to the much larger RI contrast between gas (RI ≈ 1.00) and the EW-Baker medium (RI ≈ 1.35 at 405 nm).^18^

### TEM Analysis Reveals Impurities in 10% EW-Sigma

Our previous TEM investigation of 10% EW-Baker encapsulated in GLCs identified a distinct class of mesoscopic clathrate hydrate structures.^18,19^ These structures are composed of crystalline water frameworks that encapsulate a high density of gas-filled cavities, and their arrangements differ significantly from those found in conventional gas clathrates (gas hydrates).^18,19^ Given that crystalline water is the dominant component, the RI of these structures is expected to approximate that of ice. This likely accounts for the dim nanoparticle signals observed in NTA measurements of 10% EW-Baker and their corresponding RI value. In contrast, DLS and NTA analyses of EW-Sigma (Figures 1 and 2) reveal strongly light-scattering entities, suggesting the presence of impurities within the EW mixture. To investigate this, we prepared 10% EW-Sigma samples sandwiched in GLCs and performed TEM imaging to determine whether the observed nano-objects contain contaminants. TEM micrographs (Figure 3a,b) reveal numerous unidentified mesoscopic structures with morphologies markedly distinct from the mesoscopic clathrate hydrate structures previously characterized in 10% EW-Baker. Notably, these unknown structures were absent in control samples, including bare graphene, GLCs containing pure degassed water, and those with gas-supersaturated water.^17–19^ Although mesoscopic clathrate hydrate structures are also present in 10% EW-Sigma (Figure 3c), their identification is hindered by the abundance of these unknown entities. We hypothesize that these unidentified structures may correspond to the impurities responsible for the pronounced light scattering observed in SLS and NTA measurements.

**Figure 3:**
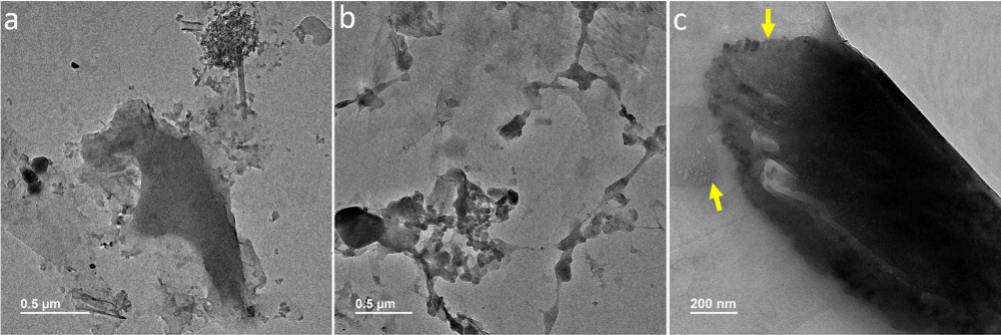
TEM of 10% EW-Sigma sandwiched in GLCs. (a and b) Unknown structures of impurities with irregular morphologies. (c) Regions with clathrate hydrate structures are indicated with yellow arrows.

### Effect of the Storage Container on Ethanol Purity

We observed that ethanol purchased from Sigma-Aldrich was supplied in a plastic (HDPE) bottle, whereas ethanol from Baker was packaged in a glass bottle. To evaluate whether the plastic container contributes to contamination in the Sigma-Aldrich ethanol, we transferred approximately 30 mL of ethanol from the original plastic bottle into a clean glass bottle (50 mL) and stored it for over 3 weeks. A small amount of this ethanol was then used to prepare 10% EW mixtures, which were encapsulated in GLCs for TEM analysis. TEM imaging revealed mesoscopic clathrate hydrate structures with morphological characteristics identical to those previously observed in 10% EW-Baker.^18^ Only a minimal presence of unidentified mesoscale structures was detected. Bright-field images of several clathrate structures recorded at underfocus and overfocus (Figure 4a,b) show a high density of nanometer-scale white spots at underfocus, which appear as dark spots at overfocus—consistent with phase contrast behavior.^17,18^ Selected area electron diffraction (SAED) from the clathrate structure marked in Figure 4a (Figure 4c) yielded diffracted spots with a *d*-spacing of 2.9 Å. The corresponding dark-field image (Figure 4d), obtained from the circled diffraction spot, revealed honeycomb-like architectures with bright features outlining the nanoscale cells. Figure 5a–c presents three distinct in-zone diffraction patterns recorded from the clathrate structure indicated by the blue arrow in Figure 4a, acquired by tilting the sample to appropriate zone axes. These patterns correspond well to Type **α**, **γ**, and **ε** diffraction patterns previously reported in 10% EW-Baker and gas-supersaturated water.^19^ The significant reduction in unidentified structures in EW mixtures prepared using ethanol (Sigma-Aldrich) that had been stored in glass for several weeks suggests that the unknown entities observed in Figure 3 might be nanoscale contaminants originated from the plastic bottle wall.

**Figure 4:**
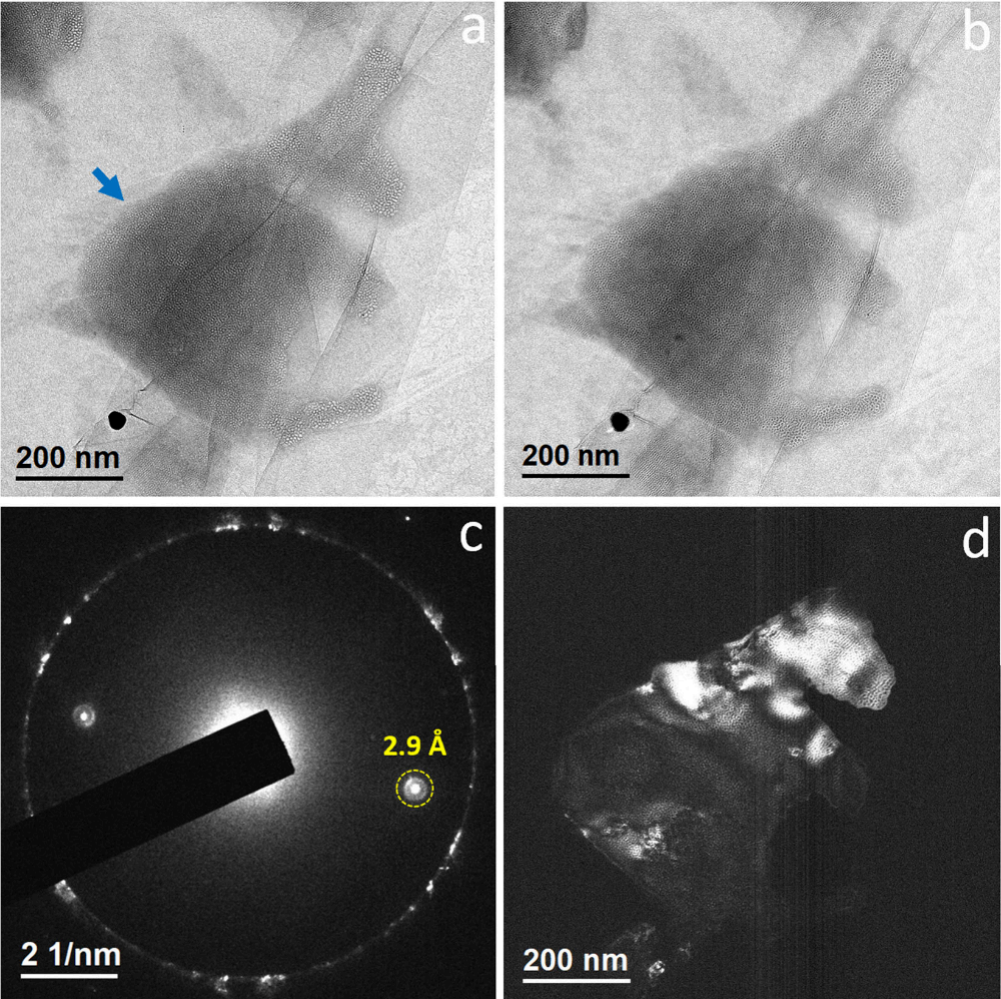
TEM analysis of 10% EW-Sigma encapsulated in GLCs following prolonged ethanol storage in a glass container. (a) Bright-field TEM image acquired under defocus conditions. (b) Bright-field TEM image acquired with overfocus. (c) SAED pattern obtained from the clathrate hydrate structure marked by an arrow in panel a. The outer ring corresponds to first-order diffraction spots of multilayer graphene, exhibiting a *d*-spacing of approximately 2.14 Å. (d) Dark-field TEM image acquired from the diffraction spot highlighted by the dashed yellow circle in panel c. Bright regions indicate crystalline domains contributing to the diffracted beam.

**Figure 5:**
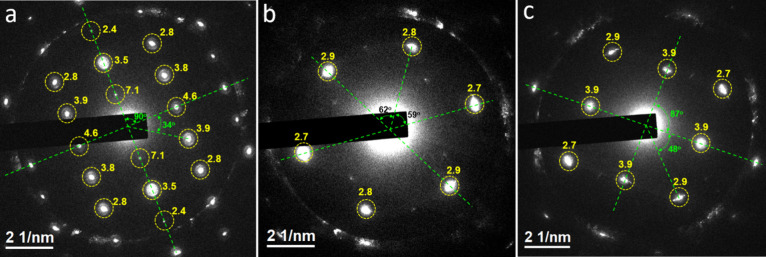
Three different in-zone diffraction patterns: (a) type α, (b) type γ, and (c) type ε. The yellow numbers adjacent to certain diffraction spots denote the corresponding *d*-spacing values in angstroms.

### Further Discussion

The above DLS, NTA, and TEM analyses indicate that ethanol from Sigma-Aldrich may contain dissolved, unidentified molecules originating from the HDPE storage container. HDPE containers are commonly manufactured with intentional additives (such as stabilizers, plasticizers, antioxidants, and processing aids) to improve their mechanical strength, durability, and resistance to environmental stress,^20,21^ and some of these additives are more soluble in ethanol than polyethylene. It is therefore highly likely that the impurities detected in the ethanol are leached additives from the HDPE container. When the ethanol is mixed with water to prepare 10% EW or 20% EW solutions, the additive molecules appear to aggregate into irregularly shaped nanoparticles, tens to hundreds of nanometers in size, exhibiting strong light-scattering signals. Purification of the ethanol effectively eliminates these contaminants, as evidenced by the lack of light-scattering particles in SLS measurements of 20% EW-distilled Sigma (Figure 1). Furthermore, prolonged storage of the ethanol in a glass bottle resulted in a significant reduction in impurity concentration. This observation suggests that a substantial fraction of the additive molecules may have been removed via adsorption onto the glass surface.

There has been serious debate about whether the mesoscopic inhomogeneities in ethanol–water mixtures observed with light scattering techniques are impurities or gas nanobubbles. Research groups advocating for the existence of gas nanobubbles primarily cite experimental evidence showing that mesoscopic structures are closely linked to the gas content in solutions—for instance, degassing often results in the disappearance of these structures.^5–7^ Some research groups advocate for impurities or contaminants because no light scattering objects are seen in aqueous solutions of ethanol that is purified prior to mixing with water.^10–12^ In addition, several experiments have indicated that the mass density of the mesoscopic structures in ethanol–water mixtures is close to that of water or ice and deviates strongly from the mass density of gas bubbles (close to zero), contradicting the picture of gas nanobubbles.^11–13^ Our studies are consistent with the above experimental observations, but a new picture has emerged. The mesoscopic clathrate hydrate structures observed with TEM are gas-containing nano-objects with a mass density close to that of water ice, explaining the observations and may resolve the puzzles about mesoscopic structures in EW mixtures. Impurities in ethanol indeed play a major role in the EW mixtures containing strongly light-scattering mesoscopic structures. If ethanol of sufficient purity is used or if ethanol is purified before the mixing of water with ethanol, strongly light-scattering objects are rarely seen and, instead, a high concentration of dim nanoparticles can be detected with NTA under proper conditions. These dim nanoparticles could be effectively removed through degassing or mixing degassed water with degassed ethanol,^18^ and might be the mesoscopic clathrate hydrate structures observed in EW mixtures sandwiched in GLCs with TEM. The mesoscopic clathrate hydrate structures are mainly composed of crystalline water molecules,^18,19^ which is expected to have an RI close to that of water ice (RI = 1.31) and consistent with our earlier measurements of RI of ∼ 1.27.^18^ The presence of the mesoscopic clathrate structures also explains many long-standing puzzles related to EW mixtures, such as a sharp decrease in excess entropy and enthalpy,^22^ a sharp increase in excess heat capacity,^23^ a sharp increase in the strength of hydrogen bonds^7,24,25^ with increasing ethanol concentration in the low ethanol concentration regime.

Many methods other than mixing water with alcohol have been reported to produce aqueous solutions containing bulk nanobubbles.^1–3,26^ As with EW mixtures, there remains considerable debate over whether these solutions truly contain nanobubbles or merely impurities.^1–3,12,14,15,26–31^ One such method is compression–decompression.^27–29^ Jaramillo-Granada et al. subjected krypton and xenon dissolved in water to pressures of up to 15 bar, then decompressed the system.^30^ Using DLS, they observed mesoscopic-scale structures. Notably, these structures persisted even after centrifugation under high gravitational fields, suggesting that their mass density closely matches that of liquid water—implying they are not gas bubbles. The authors proposed that these entities may be clathrate-hydrate nanostructures, a hypothesis consistent with our earlier TEM observations of mesoscopic clathrate hydrate structures in gas-supersaturated water confined within GLCs, where the water was similarly prepared via compression–decompression.^17^ More recently, Iqbal et al. reported that the compression–decompression method does not generate bulk nanobubbles within the pressure range of 1–150 bar.^26^ Their conclusion was based on the detection of very low scattering intensity in CO_2_-supersaturated water, as measured by both DLS and Tyndall scattering. However, these measurements do not exclude the possibility of a high concentration of weakly scattering objects with small dimensions (on the order of tens of nanometers) as we have presented in this work. Further insight comes from two studies employing attenuated total reflectance infrared spectroscopy on water samples containing bulk “nanobubbles”.^29,31^ These investigations revealed the presence of hard hydrogen bonds—comparable to those found in ice and gas hydrates. It was proposed that such bonds exist at the surfaces of nanobubbles, potentially reducing gas diffusivity across the interfacial film and contributing to nanobubble stability.^29,31^ Alternatively, these hard hydrogen bonds may reflect strengthened hydrogen bonding within mesoscopic clathrate hydrate structures.^17–19^

## Conclusion

There has been ongoing debate regarding whether the mesoscopic structures observed in ethanol–water (EW) mixtures via light scattering techniques are genuine gas bubbles or merely impurities. Our current study reveals that the scattering intensity of these structures—whether strong or weak—depends critically on the purity of the ethanol used prior to mixing. Structures that exhibit strong light scattering are likely attributable to impurities in the ethanol. These could not be effectively removed by degassing alone but were almost entirely eliminated through ethanol purification methods such as distillation. One plausible source of these impurities is additive molecules leached from plastic (HDPE) containers into the ethanol. Upon mixing with water, these molecules may aggregate into mesoscopic particles, contributing to the observed scattering. In contrast, the dimly scattering particles were effectively removed by degassing, either before or after mixing ethanol with water. Their presence appears to be associated with gas supersaturation in the solution. Based on our findings, we propose that these dim, colloidal-like particles are nanoscale gas–water composite structures—most likely mesoscopic clathrate hydrate structures, as previously observed by transmission electron microscopy (TEM).

## Supplementary Material





## Notes

DLS measurements of the particle size distribution of 20% EW-Sigma (Figure S1), comparison of NTA images of 10% EW-Sigma and 10% EW-Baker acquired at two camera levels (Figure S2), and camera levels of the NanoSight NS500 and the corresponding gain and shutter time (Supporting Table 1) (PDF)
NTA video of 10% EW-Baker acquired at camera level 16 (Supporting Video 1) (MP4)
