# Supplementary material for: Mesoscopic Inhomogeneities in Ethanol–Water Mixtures: Are They Nanobubbles, Impurity Aggregates, or Nanoscale Gas–Water Composite Structures?
Source: Langmuir. 2026 Feb 10;42(7):5452–8. doi: 10.1021/acs.langmuir.5c05369 (PMC12937110; doi:10.1021/acs.langmuir.5c05369)
Supplement: Supplementary file 1 [file la5c05369_si_001.pdf]

Supporting Information

Mesoscopic Inhomogeneities in Ethanol–Water Mixtures: Are They Nanobubbles, Impurity Aggregates, or Nanoscale Gas–Water Composite Structures?

Chien-Chun Chen[a], Wei-Hao Hsu[a], Chun-Jen Chen[a], Tzu-Chieh Yen[a], Ching-Hsiu Chen[a], C. K. Chan[a], Che-Ming Jack Hu[b], and Ing-Shouh Hwang[*a]

[a] Institute of Physics, Academia Sinica, Nankang, Taipei 115, Taiwan

[b] Institute of Biomedical Sciences, Academia Sinica, Taipei 115, Taiwan

Email: ishwang@phys.sinica.edu.tw

1. Supporting Figures:

Figure S1: Dynamic light scattering (DLS) measurements of particle size distribution of 20% EW-Sigma.

Figure S2: Comparison of nanoparticle tracking analysis (NTA) images of 10% EW-Sigma and 10% EW-Baker acquired at two camera levels.

2. Supporting Table:

Supporting Table 1: Camera levels of the NanoSight NS500 and the corresponding gain and shutter time.

3. Supporting Video:

Supporting Video 1: NTA video of 10% EW-Baker acquired at camera level 16.

1. Supporting Figures

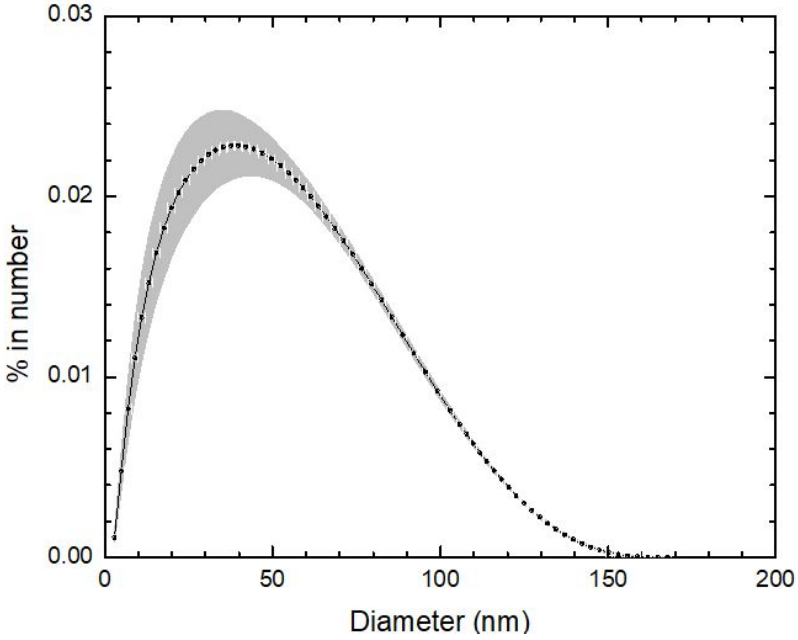

Figure S1. DLS measurements of particle size distribution of 20% EW-Sigma. The viscosity of 20% EW mixture was assumed to be 1.8 cP [I. S. Khattab et al., Korean J. Chem. Eng. 29, 812 (2012)].

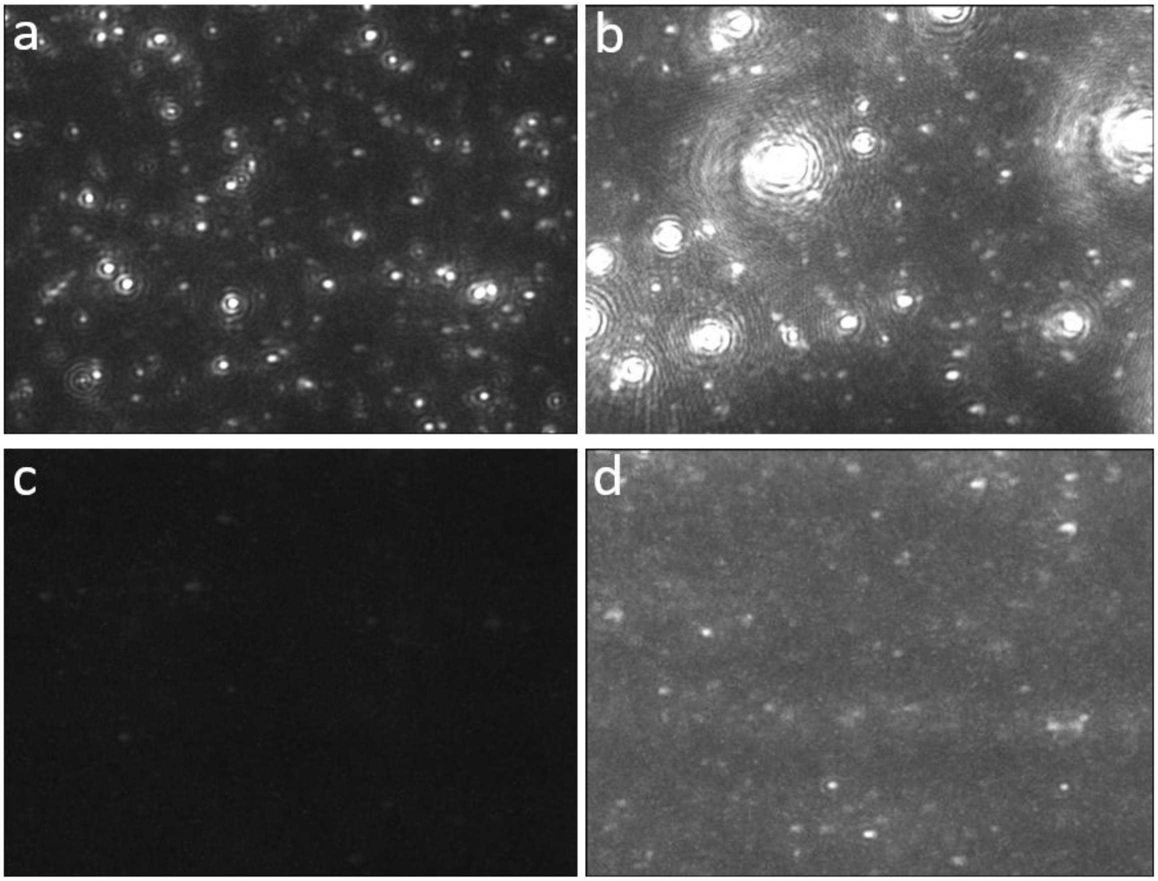

Figure S2. Comparison of NTA images of 10% EW-Sigma and 10% EW-Baker acquired at two camera levels. (a) 10% EW-Sigma; camera level of 14. (b) 10% EW-Sigma; camera level of 16. Many particles were saturated at this camera level. (c) 10% EW-Baker; camera level of 14. The camera level was too low to see all particles clearly. (d) 10% EW-Baker; camera level of 16.

2. Supporting Table

Level	Gain	Shutter (ms)
5	15	1.12
6	15	2.15
9	15	15.18
10	73	17.40
12	146	30.00
13	219	30.80
14	366	31.48
15	366	30.15
16	512	32.50

Supporting Table 1 Camera levels of the NanoSight NS500 and the corresponding gain and shutter time.
